# Metabolic Dysfunction Associated Steatotic Liver Disease and Bone Turnover Markers in Postmenopausal Women: A Cross‐Sectional Study in Central Nepal

**DOI:** 10.1002/hsr2.71777

**Published:** 2026-02-19

**Authors:** Sunita Maharjan, Pragyan Dahal, Bishal Ranabhat, Jyotsna Shakya

**Affiliations:** ^1^ Manmohan Memorial Institute of Health Sciences Kathmandu Nepal; ^2^ Grande International Hospital Kathmandu Nepal

**Keywords:** bone markers, MASLD, post menopause

## Abstract

**Background and Aims:**

Metabolic dysfunction‐associated steatotic liver disease, a condition of fatty infiltration in the liver, is mostly observed in women. Women after menopause are more likely to develop insulin resistance, hyperlipidemia, and visceral fat storage, which are all recognized to be risk factors for MASLD. Chronic low‐grade inflammation in hepatocytes, which is a hallmark of MASLD, may significantly affect bone metabolism. This study aimed to evaluate the relationship between MASLD and bone markers in postmenopausal women.

**Methods:**

The study is a cross‐sectional study, carried out in Manmohan Memorial Teaching Hospital, Kathmandu from March 2019 to August 2019. Altogether 105 cases of MASLD and non‐MASLD postmenopausal women were included in this study as case and control respectively. Anthropometric measurements were recorded using a standard checklist. Serum calcium, phosphorus, and alkaline phosphatase were measured, and statistical analysis was done using SPSS version 16. A *p* value < 0.05 was indicated as statistically significant.

**Results:**

The median age of postmenopausal women did not differ significantly between those with and without MASLD (Non‐MASLD: 60.0 years, IQR 53.0–69.0; MASLD: 58.0 years, IQR 55.0–65.0; *p* = 0.66). Similarly, systolic and diastolic blood pressures (SBP and DBP) showed no significant differences between the groups. Women with MASLD, however, exhibited higher anthropometric measurements, including body mass index (BMI: 26.4 vs. 20.3 kg/m²) and waist circumference (WC: 96.5 cm vs. 88.9 cm). Biochemically, median serum calcium levels were significantly lower in women with MASLD (8.9 mg/dL, IQR 8.1–9.7), while median serum phosphorus (4.9 mg/dL, IQR 4.3–6.3) and alkaline phosphatase (ALP) levels (197.8 U/L, IQR 166.7–247.5) were significantly higher compared to the non‐MASLD group (*p* < 0.001).

**Conclusion:**

This study showed significant changes in bone markers (serum calcium, phosphorus, and ALP) in postmenopausal women with MASLD than those without MASLD. Throughout our study, confounding factors such as BMI and waist circumference have significantly influenced the occurrence of bone disorders.

## 1 Introduction

Metabolic Dysfunction‐Associated Steatotic Liver Disease (MASLD) is a condition marked by considerable lipid accumulation (5%–10%) in hepatic tissue in the absence of excessive alcohol use, prolonged use of a steatogenic medication and monogenic hereditary disorders, diagnosed with imaging or histology and with presence of one or more of the cardiometabolic risk factors (obesity, type 2 diabetes mellitus, hypertension and dyslipidemia such as elevated triglyceride (TG) and decreased high‐density lipoprotein cholesterol (HDL‐C) levels) [1, 2, 3, 4, 5]. MASLD has a wide range of clinical manifestations, from simple steatosis to nonalcoholic steatohepatitis (NASH), which can progress to cirrhosis, hepatic fibrosis, and ultimately hepatic carcinoma [6]. Metabolic syndrome, which is both the cause and the result of primary MASLD, is mutually and reciprocally correlated with it [7]. Similarly, the use of various medications, exposure to pollutants, surgeries for obesity, and total parenteral nutrition can contribute to secondary MASLD [8].

Women between the ages of 40 and 61 experience menopause, which is the irreversible end of the menstrual cycle brought on by a decline in ovarian follicular activity [9, 10, 11]. Menopause occurs naturally or is accompanied by surgery, chemotherapy, or radiation at any age [12]. After menopause, estrogen production gradually declines, which leads to decreased calcium levels while raising alkaline phosphatase levels [13, 14]. This results in the loss of minerals from bone and makes postmenopausal women more prone to bone disorders. The most prevalent bone condition in postmenopausal women is osteoporosis, which is defined by reduced bone mass and structural bone tissue degradation [14, 15].

Women after menopause are more likely to develop insulin resistance, hyperlipidemia, and visceral fat accumulation, all of which are known risk factors for MASLD [16]. MASLD and osteoporosis have been related in recent investigations; however, the pathophysiological processes are not fully understood. There are numerous viable explanations. First, excessive hepatic fat accumulation causes low‐grade, chronic inflammation that contributes to bone loss, osteoporosis, and an increased risk of fractures. Experimental studies have provided substantial evidence suggesting that specific inflammatory cytokines play a significant role in the pathophysiology of bone loss or osteoporosis [17, 18, 19]. In addition, MASLD has a significant impact on the development of oxidative stress, which is directly linked to the occurrence of osteoporotic fracture [20, 21].

Different bone markers can help to identify the risk of bone disorders in postmenopausal women. Serum bone alkaline phosphatase is one of the most sensitive and specific bone markers that reflect the accelerated turnover associated with bone destruction of aging, menopause, and various conditions affecting bone metabolism [22]. Macro minerals like calcium and phosphorus also help to identify bone status. Biochemical bone markers such as calcitonin, vitamin D, serum osteocalcin, urinary hydroxyproline, procollagen I extension peptides, etc can be performed for the diagnosis of osteoporosis [23, 24].

Limited studies have shown that there is a low level of Vitamin D in MASLD patients. Low 25(OH)‐D levels, elevated PTH (secondary hyperparathyroidism), and elevated blood alkaline phosphatase are signs of vitamin D deficiency. During vitamin D deficiency, calcium‐binding proteins (calmodulin) are decreased which leads to decreased intestinal absorption of calcium and results in hypocalcemia. Due to the reciprocal link between calcium and phosphorus, hypocalcemia causes a rise in phosphorus levels [21]. Despite a few limited studies conducted on MASLD in Nepal, a study on postmenopausal women and its relationship to bone markers had been conducted for the first time in Nepal. Therefore, the prime objective of this study was to observe the level of serum calcium, phosphorus, and ALP in postmenopausal women with and without MASLD. As postmenopausal women are already prone to bone disorders, presence of MASLD further increases the burden on them. Hence, this study may also help in the early detection of bone disorders and can aid in better treatment.

## 2 Materials and Methods

### 2.1 Study Area and Design

A cross‐sectional study conducted at the Manmohan Memorial Teaching Hospital (MMTH), a tertiary care facility in Kathmandu, Nepal (27.71°N, 85.31°E). MMTH is a 300‐bedded hospital with a total area sprawling over 5000 sq. ft. The study was conducted in a tertiary care hospital in Nepal within the Department of Biochemistry in conjunction with the Department of Radiology over 6 months (March 2019 to August 2019).

### 2.2 Study Population and Sampling

This study employed a cross‐sectional design, in which all clinical information, ultrasonography findings, and blood samples were collected once during the participants' routine clinical visits. No follow‐up assessments or longitudinal outcome measurements were performed. A total of 210 postmenopausal women were enrolled over a 6‐month period, comprising 105 women with MASLD and 105 women without MASLD. MASLD was diagnosed by radiologists using abdominal ultrasonography.

The exclusion criterion was as follows: Blood samples of women, who had amenorrhea associated with a hysterectomy and other conditions such as a history of diabetes mellitus, rheumatoid arthritis, bone‐related medications, hormone replacement therapy, thyroid disorders, renal diseases, jaundice, and liver diseases, as well as women who had smoking and alcohol intake lifestyles were excluded from the study.

The sample size for this cross‐sectional study comparing two independent groups of postmenopausal women (MASLD and non‐MASLD) was calculated using the formula for comparing two proportions:

$$N=\frac{{\left(Z_{\alpha /2}+Z_{\beta }\right)}^{2}[p_{1}(1-p_{1})+p_{2}(1-p_{2})]}{{\left(p_{1}-p_{2}\right)}^{2}}$$

where $Z_{\alpha /2}=1.96$ corresponds to a 95% confidence level (*α* = 0.05) and $Z_{\beta }=0.84$ corresponds to 80% statistical power (*β* = 0.20). The expected difference in proportions was 0.20, with $p_{1}=0.579$ representing the prevalence of MASLD among postmenopausal women and $p_{2}=0.375$ representing the prevalence among non‐MASLD postmenopausal women, based on previous studies [25, 26]. Using these parameters, the minimum required sample size was calculated as 93 participants per group. To account for potential sample loss due to mislabeling, breakage, or spillage, a 13% allowance was added. Therefore, the final estimated sample size was found to be 105 participants of post‐menopausal women for each group (MASLD and non MASLD).

### 2.3 Experimental Procedure

A cross‐sectional study was conducted over a period of 6 months among the postmenopausal women of Manmohan Memorial Teaching Hospital (MMTH), Kathmandu, Nepal. The selection of 210 postmenopausal women was based on ultrasonography. Among them, 105 were postmenopausal women with MASLD, and 105 were non‐ MASLD postmenopausal women. Information regarding the patient demography (age, BMI, waist circumference, blood pressure), duration of menopause, age of menarche, history of hysterectomy, bone‐related medication intake, and any hormonal therapy were collected and recorded in a clinical profile form.

For the determination of biochemical parameters, approximately 5 mL of venous blood was taken from each woman. The levels of serum calcium, phosphorus, and alkaline phosphatase (ALP) were determined to serve as biochemical markers of bone turnover. Serum calcium, serum phosphorus, and ALP were measured using a semi‐automatic analyzer (Accurex Biomedical Pvt. Ltd., Mumbai, India). The reference range for serum calcium was considered as 8.5–11.0 mg/dL, serum phosphorous was 2.5–5.0 mg/dL, and normal activity of serum ALP was 108–306 IU/L [27].

Anthropometric variables and clinical characteristics were recorded using a standard checklist. Standing upright barefoot, the subjects' heights were measured with a wall scale meter. A standard digital weighing scale was used to assess the patient's weight while they were barefoot and wearing only the most basic of clothing. Body mass index (BMI) was calculated and expressed in kg/m^2^. Patients who had previously been diagnosed with hypertension by a clinician, had a systolic blood pressure of 140 mmHg or above, a diastolic blood pressure ≥ 90 mmHg, or were receiving an antihypertensive medication were all considered to have a history of hypertension [28].

**Figure 1:**
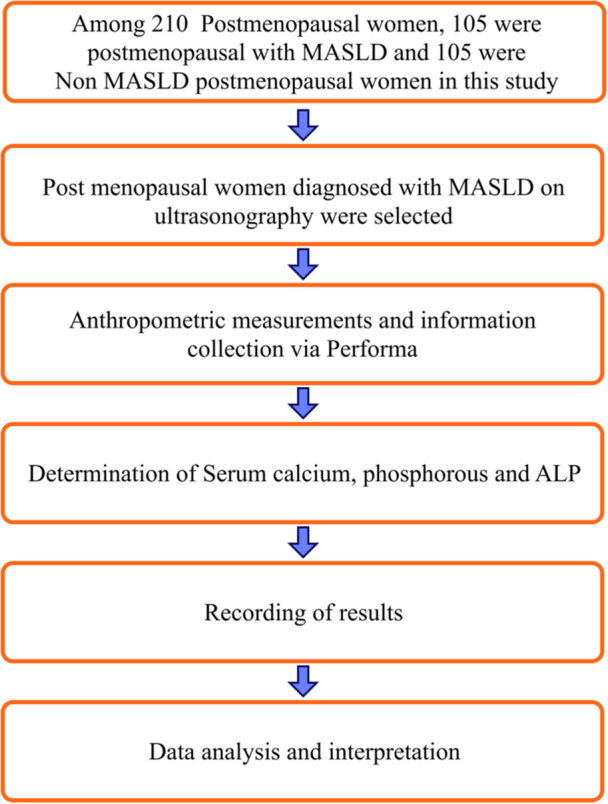
Illustration of flowchart that determines work methodology. The above diagram shows the study workflow outlining clinical assessment and biochemical profiling of MASLD in postmenopausal women.

The overview of the procedure was depicted in a flow chart (Figure 1).

### 2.4 Criteria for Grading of MASLD

Patients with visibly dispersed hepatic steatosis were further graded to characterize the degree of fatty alteration in the liver based on ultrasonography and histologic nomenclature. Grading was done based on the standard criteria accepted by the American, Gastroenterological Association [29, 30].

Grade I (mild): elevated hepatic echogenicity accompanied by visible diaphragmatic and periportal echogenicity.

Grade II (moderate): elevated hepatic echogenicity without diaphragm obscuration, accompanied by undetectable periportal echogenicity.

Grade III (severe): hepatic echogenicity increased along with subtle periportal echogenicity and diaphragm obscuration.

### 2.5 Statistical Analysis

Data was analyzed using SPSS version 16 (IBM Corp., Armonk, NY, USA) and Microsoft Excel 2013. The Shapiro–Wilk test was employed to assess the normality of the data. As the data were not normally distributed, the Mann–Whitney *U* test was used to compare biochemical and anthropometric parameters between postmenopausal women with and without MASLD. *χ* ^2^ test was used to compare difference between BMI category between MASLD and non‐MASLD. For comparisons among multiple groups (non‐MASLD, MASLD grade I, and grade II), the Kruskal–Walli's test was applied. The *p* value < 0.05 was considered statistically significant.

## 3 Results

### 3.1 Demographic, Anthropometric, and Biochemical Characteristics by MASLD Status

The median age of postmenopausal women did not differ significantly between the MASLD and non‐MASLD groups (58.0 years (IQR 55.0–65.0) vs. 60.0 years (IQR 53.0–69.0); *p* = 0.66), indicating comparable demographic characteristics. Similarly, systolic and diastolic blood pressures were not significantly different between groups (SBP: 128 vs. 126 mmHg, *p* = 0.38; DBP: 79 vs. 78 mmHg, *p* = 0.67). In contrast, women with MASLD had significantly higher body mass index (26.4 vs. 20.3 kg/m²; *p* = 0.04) and in waist circumference (96.5 vs. 88.9 cm; *p* < 0.001), reflecting increased overall and central adiposity. Biochemically, MASLD was associated with lower median serum calcium levels (8.9 vs. 9.4 mg/dL; *p* < 0.001) and higher serum phosphorus (4.9 vs. 4.0 mg/dL; *p* < 0.001) and alkaline phosphatase (ALP) levels (197.8 vs. 171 U/L; *p* < 0.001) compared to non‐MASLD participants. These findings indicate that postmenopausal women with MASLD exhibit greater adiposity and altered metabolic and hepatic parameters, whereas age and blood pressure remain similar between groups. The results are summarized in Table 1 below.

**Table 1:** Comparison of demographic, anthropometric, and biochemical variables in postmenopausal women with and without MASLD.

Variable	Non‐MASLD (*n* = 105)	MASLD (*n* = 105)	*p* value (Mann–Whitney U)
Age (years), median (IQR)	60.0 (53.0–69.0)	58.0 (55.0–65.0)	0.66
BMI (kg/m²), median (IQR)	20.3 (22.5–26.8)	26.4 (24.8–29.1)	0.04
Waist circumference (cm), median (IQR)	88.9 (82.0–95.0)	96.5 (90.0–103.0)	< 0.001
SBP (mmHg), median (IQR)	126 (115–136)	128 (116–140)	0.38
DBP (mmHg), median (IQR)	78 (72–85)	79 (72–86)	0.67
Serum calcium (mg/dL), median (IQR)	9.4 (8.9–9.9)	8.9 (8.1–9.7)	< 0.001
Serum phosphorus (mg/dL), median (IQR)	4.0 (3.7–4.3)	4.9 (4.3–6.3)	< 0.001
ALP (U/L), median (IQR)	171 (150–200)	197.8 (166.7–247.5)	< 0.001

### 3.2 Comparison of Biochemical Parameters and Anthropometric Variables in Postmenopausal Women With Different Grades of MASLD and Non‐MASLD by Applying the Kruskal–Wallis Test

**Figure 2:**
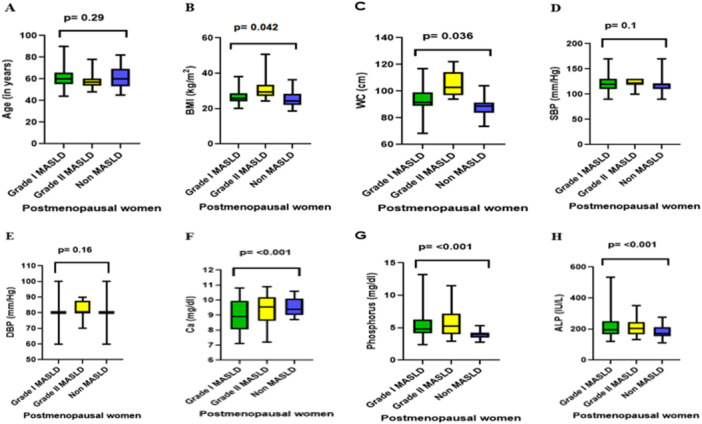
Comparison of anthropometric and biochemical parameters in postmenopausal women according to MASLD status. Box plots show median values and interquartile ranges for postmenopausal women with Grade I MASLD, Grade II MASLD, and non‐MASLD groups. (A) Age (years), (B) body mass index (BMI, kg/m²), (C) waist circumference (WC, cm), (D) systolic blood pressure (SBP, mmHg), (E) diastolic blood pressure (DBP, mmHg), (F) serum calcium (Ca, mg/dL), (G) serum phosphorus (mg/dL), and (H) alkaline phosphatase (ALP, U/L). Statistically significant differences were observed for BMI, WC, Ca, phosphorus, and ALP (*p* < 0.05), whereas age and blood pressure did not differ significantly.

Anthropometric and biochemical parameters were compared among Grade I MASLD, Grade II MASLD, and non‐MASLD postmenopausal women (Figure 2). There was no significant difference in age across the groups (*p* = 0.29). Both BMI and waist circumference were significantly higher in Grade II MASLD compared to non‐MASLD women (BMI: *p* = 0.042; WC: *p* = 0.036), with Grade I MASLD showing intermediate values. Systolic and diastolic blood pressures did not differ significantly among the groups (SBP: *p* = 0.10; DBP: *p* = 0.16). Regarding biochemical markers, serum calcium was significantly higher in Grade II MASLD compared to non‐MASLD women (*p* < 0.001). Serum phosphorus and alkaline phosphatase (ALP) levels were also significantly elevated in MASLD groups relative to non‐MASLD women (phosphorus: *p* < 0.001; ALP: *p* < 0.001), with the highest values observed in Grade II MASLD. These findings (Figure 2) indicate that postmenopausal women with MASLD, particularly those with more advanced disease, exhibit increased BMI, central adiposity, and elevated bone turnover markers, while age and blood pressure remain comparable across groups.

## 4 Discussions

Metabolic Dysfunction‐Associated Steatotic Liver Disease (MASLD), a condition of fatty infiltration in the liver, is most commonly observed in women [31]. MASLD not only affects the liver but also increases the risk of extrahepatic disease conditions like osteoporosis [32]. However, there are inconsistent findings about the association between different grades of MASLD and bone markers. Hence, this study aimed to compare the relationship between MASLD and bone markers in postmenopausal women with and without MASLD.

Our study has shown that serum calcium level was significantly decreased while phosphorus and ALP were significantly increased in postmenopausal women with MASLD when compared to non‐MASLD postmenopausal women. In contrast, no significant difference in calcium and phosphorus was observed in postmenopausal women with NAFLD in the study conducted among Caucasian postmenopausal women with NAFLD and Type II Diabetes by Mantovani et al. [33]. This type of result may be caused due to the different study populations and geographical variations.

According to a study conducted in Nepal, Serum ALP level was shown to be significantly increased in NAFLD patients as compared to non‐ NAFLD which is concordant with our study [7, 34]. Our study has shown that there was a significant difference in BMI and waist circumference values whereas no significant difference in SDP and DBP of NAFLD patients in comparison to the control group. In the study, NAFLD patients had significantly higher BMI and waist circumference values when compared to the control group [7]. Similarly, there was a significant difference in BMI but no significant difference in SDP and DBP in the study conducted among Caucasian postmenopausal women with NAFLD and type II diabetes [33].

Many proteins are synthesized from the liver which is involved in bone metabolism and is the regulator of several bone metabolism pathways like Vitamin D metabolism pathway. Since MASLD is the most common cause of chronic liver disease, it affects bone metabolism which leads to changes in bone markers [34]. Several studies have shown that Vitamin D was significantly decreased in NAFLD patients [33, 35]. Decreased Vitamin D levels may interfere with the absorption of calcium and hence may decrease serum calcium levels. As calcium is inversely proportional to phosphorus; low calcium causes the increased level of phosphorus. Similarly, alkaline phosphatase may get elevated in a Vitamin D‐deficient state [36].

The level of vitamin D affects the rate at which bones break down, the total mineralization of the skeleton, and the likelihood of fractures. Research on vitamin D supplementation that improves vitamin D status has shown reductions in bone turnover, an increase in bone mineral density, and a drop in the incidence of fractures [37].

Plenty of studies have employed a variety of methodologies and biochemical markers to try and characterize changes in bone mineralization in diabetic subjects since Albright and Reifenstein first documented the incidence of osteoporosis in patients with poorly managed diabetes in 1948. Decreased bone mineral density and serum osteocalcin, but elevated levels of urine hydroxyproline and serum alkaline phosphatase were observed in diabetic patients. Patients with diabetes nephropathy (DN) showed elevated circulation phosphate levels and decreased urine phosphate excretion, whereas patients with group D (Diabetes) showed lower circulating phosphate levels and increased urinary phosphate excretion. Hence, individuals with diabetes exhibit abnormal bone mineral metabolism [38].

In the present study, body mass index (BMI) was higher in postmenopausal women with MASLD than those without. If BMI is ≥ 30 kg/m^2^ then it is defined as obesity. Similarly, BMI of 25–29.9 kg/m^2^ and < 18.5 kg/m^2^ are considered as overweight and underweight respectively [39]. Out of 105 postmenopausal women with MASLD, 52% were overweight while 32% were obese in our study. In contrast to other corresponding groups, those with a BMI of 25 or above had a higher incidence of osteoporosis [40].

Obesity is associated with a significant increase in serum leptin and a decrease in adiponectin. Furthermore, excessive leptin secretion and/or decreased adiponectin production by adipocytes in obesity may have an impact on bone formation and directly or indirectly affect bone resorption through increased production of pro‐inflammatory cytokines. Similarly, a high‐fat diet, often a cause of obesity, has been reported to interfere with intestinal calcium absorption. Free fatty acids can result in unabsorbable, insoluble calcium soaps, which lower calcium availability for bone growth and calcium absorption [41]. Excessive fat accumulation causes the severity of fatty liver which may increase the risk of osteoporosis [42].

### 4.1 Limitations and Strengths of the Study

Our study had several limitations. First, the current study is a single‐centered study due to which we had a limited sample size of MASLD concerned to this selective study for postmenopausal women therefore; a multicentered large prospective cohort study would be required to draw more sample size and precise findings. In addition, we were unable to measure hormonal levels, vitamin D, and other bone markers of osteoporosis such as serum osteocalcin, urinary hydroxyproline, procollagen I extension peptides, etc in limited resource settings due to the unavailability of funds.

After menopause, there is a progressive decline in estrogen synthesis, which adds to aberrant fat distribution marked by a build‐up of visceral fat. The severity of fatty liver is brought on by excessive fat storage, which may increase the risk of osteoporosis. In postmenopausal women, decreased levels of estrogen are considered to be responsible for increasing bone resorption and decreasing osteoblast differentiation activity [42, 43]. But due to the lack of funding, we were unable to estimate estrogen level which may aid in diagnosis of osteoporosis. Likewise, postmenopausal women are included in our study; however, there is lack of information regarding the menopause's frame time.

Medication that is known to cause both steatosis and steatohepatitis can be categorized into three main categories: medications that cause both conditions separately (such as amiodarone and perhexiline maleate); medications that can trigger latent NASH (such as tamoxifen); and medications that cause sporadic episodes of steatosis/steatohepatitis (such as carbamazepine) [44]. Insulin sensitizers, such as pioglitazone and rosiglitazone, along with lipid‐lowering medications including fibrates and statins, and other agents like betaine, *N*‐acetylcysteine, *α*‐tocopherol, and metformin, have been shown to alleviate fatty liver [45]. While this study did not focus on these medications, it is important to acknowledge that they may influence bone markers, potentially impacting the results.

During menopause, adopting lifestyle changes such as maintaining a healthy diet, engaging in regular physical activity, and achieving weight loss is crucial for effective management of MASLD. These therapies are effective in improving liver function and lowering the amount of fat in the liver [46, 47, 48, 49]. However, the study did not address the modifications in lifestyle following menopause. Despite we had some limitations, our study possesses certain strengths. This type of study about bone markers in postmenopausal women with MASLD was conducted for the first time in Nepal. Our investigated findings provide valuable and concise information to the treating clinicians and the data availability of bone markers in postmenopausal women is very limited in the literature. Patients having established osteoporosis, significant comorbidities (such as renal impairment, thyroid/parathyroid disorders, diabetes mellitus, and liver diseases), or those taking oral supplements (such as vitamin D or bisphosphonates) that are known to disrupt bone metabolism are excluded in our study. We think that the inclusion of patients with these illnesses may confound the interpretation of data (estimation of Serum calcium, phosphorus and ALP).

## 5 Conclusions

This study was designed to evaluate the bone markers (serum calcium, phosphorus, and alkaline phosphatase) level and their comparison in postmenopausal women with and without MASLD. Based on our study, serum calcium was significantly decreased while serum phosphorus and ALP were significantly increased in postmenopausal women with MASLD than those without MASLD. However, there was no change observed in bone markers between postmenopausal women with Grade I and II MASLD. Hence, this study concludes that postmenopausal women with MASLD are at a higher risk of developing bone disorders like osteoporosis than those without MASLD. So, the evaluation of the bone markers among postmenopausal women especially MASLD‐diagnosed women is crucial for the early diagnosis of osteoporosis. Appropriate treatment and control practices are required for the effective management of these conditions before they develop into complications.

## Author Contributions

**Sunita Maharjan:** writing original draft, conceptualization, methodology, investigation, visualization. **Pragyan Dahal:** writing – original draft, validation, writing – review and editing, formal analysis, software, resources, and investigation. **Bishal Ranabhat:** writing – review and editing, formal analysis. **Jyotsna Shakya:** supervision, validation, conceptualization.

## Funding

The authors received no specific funding for this work.

## Ethics Statement

The ethical clearance was taken from the Institutional Review Board at Manmohan Memorial Institute of Health Sciences with Ref. No: 76/75 and registration number: MMIHS‐IRC 392.

## Consent

Verbal and written consent was obtained from the participating patients.

## Conflicts of Interest

The authors declare no conflicts of interest.

## Transparency Statement

The lead author Pragyan Dahal affirms that this manuscript is an honest, accurate, and transparent account of the study being reported; that no important aspects of the study have been omitted; and that any discrepancies from the study as planned (and, if relevant, registered) have been explained.

## Data Availability

Most of the data is clearly shown in our study. Raw data will be available upon request with the first author (maharjansunita2022@gmail.com).
